# Obstacles and expectations of rare disease patients and their families in Türkiye: ISTisNA project survey results

**DOI:** 10.3389/fpubh.2022.1049349

**Published:** 2023-01-04

**Authors:** Ozden Hatirnaz Ng, Ilayda Sahin, Yucel Erbilgin, Ozkan Ozdemir, Emrah Yucesan, Nazli Erturk, Merve Yemenici, Ozlem Akgun Dogan, Sibel Aylin Ugur Iseri, Ilhan Satman, Yasemin Alanay, Ugur Ozbek

**Affiliations:** ^1^Department of Medical Biology, Acibadem Mehmet Ali Aydinlar University School of Medicine, Istanbul, Türkiye; ^2^Acibadem Mehmet Ali Aydinlar University Rare Diseases and Orphan Drugs Application and Research Center (ACURARE), Istanbul, Türkiye; ^3^Department of Medical Genetics, Acibadem Mehmet Ali Aydinlar University School of Medicine, Istanbul, Türkiye; ^4^Department of Medical Biotechnology, Institute of Health Sciences, Acibadem Mehmet Ali Aydinlar University, Istanbul, Türkiye; ^5^Department of Genetics, Aziz Sancar Institute of Experimental Medicine, Istanbul University, Istanbul, Türkiye; ^6^Department of Genome Studies, Institute of Health Sciences, Acibadem Mehmet Ali Aydinlar University, Istanbul, Türkiye; ^7^Department of Neuroscience, Institute of Neurological Sciences, Istanbul University-Cerrahpasa, Istanbul, Türkiye; ^8^Turkish Public Health and Chronic Disease Institute, Health Institutes of Türkiye, Istanbul, Türkiye; ^9^Department of Pediatrics, Acibadem Mehmet Ali Aydinlar University School of Medicine, Istanbul, Türkiye; ^10^Division of Endocrinology and Metabolism, Department of Internal Medicine, Istanbul Faculty of Medicine, Istanbul University, Istanbul, Türkiye

**Keywords:** rare disease, survey, Türkiye, ISTisNA, patient expectations

## Abstract

Rare disease patients constitute a significant part of the healthcare system of all countries. However, the information on the experiences during disease processes and daily life of rare disease patients is still limited. So far, there is a small number of studies conducted in Türkiye, and they mainly cover specific issues like education or anxiety. Here we present a comprehensive survey analysis conducted among the patients and their families within the scope of the Istanbul Solution Platform for Undiagnosed and Rare Diseases-ISTisNA project. A total of 498 individuals responded to the survey, and 58% of the participants answered all questions. The majority of the patients were in the age range of 1–10 years (44.7%), and 91% of all the patients had a precise diagnosis. The diagnosis rate in the first 6 months was 69%, and almost 10% of the patients remained undiagnosed. The mothers were the primary caregivers (72%). Nearly 30% of the caregivers had to quit their jobs and 25% of the patients (0–18 years) had to leave school. Accessing physicians with relevant specialization and reaching treatments/medications/supplements were the two main obstacles the participants mentioned, with a frequency of 81% and 73%, respectively. Around 50% of participants noted that they commonly faced difficulties at work/school and in their social lives. The highest expectation or priority was the establishment of rare disease-specific diagnosis and treatment centers, accurate and detailed information on diseases in the Turkish language, and easy access to physicians, treatments, and supportive therapies. To the best of our knowledge, this is the most comprehensive survey conducted on the rare disease community in Türkiye. These results show that regardless of the country, the individuals affected by rare diseases and their families have similar problems and expectations. On the other hand, regional and country-specific issues are still in the line to be solved. These studies can provide a deeper insight into rare diseases and guide the activities of Türkiye's national rare disease action plan.

## 1. Introduction

The definition of rare disease differs among different populations. For instance, in Europe, diseases with a prevalence of < 5 in 10.000 are considered rare (1). In the USA, this frequency is determined as < 20.000 (2) and in Japan as < 50.000 or 4 in 10.000 (3). Although Türkiye did not determine an official country-specific prevalence, the European threshold is used to define rare diseases (4). On the other hand, despite all the available tests and evaluations, some conditions remained unknown due to a lack of appropriate diagnostic tests or enough medical knowledge, and they are described as undiagnosed diseases.

The estimated number of rare diseases is between 6,000 and 8,000, affecting 8–10% of the world's population (5, 6). According to these numbers, rare diseases must affect more than 6 million individuals in Türkiye. Although rare diseases present heterogeneous clinical and genetic features, many of the social and economic obstacles are similar worldwide (7–9). Most of the patients suffer from misdiagnosis, or they even remain undiagnosed for years (10, 11). A previous survey presented the average diagnosis time for a rare disease patient as 5–7 years (12), whereas some rare diseases maybe diagnosed in a shorter time.

Rare diseases are seen more frequently in countries with increased rates of parental consanguinity. The overall consanguineous marriage rate in Türkiye is reported as 24%, ranging from 10 to 43% in different regions of the country (13). According to these findings, rare diseases must affect 5–7 million individuals in Türkiye (14). Recently, new regulations regarding rare and undiagnosed diseases have been introduced in Türkiye. In March 2020, the Grand National Assembly of Türkiye established a research commission on rare diseases, including amyotrophic lateral sclerosis (ALS), spinal muscular atrophy (SMA), Duchenne muscular dystrophy (DMD), multiple sclerosis (MS), and shared a detailed report emphasizing the needs of rare disease patients (15). The commission report stated that main needs are (i) specialized centers, (ii) national databases, (iii) a broader national health service, (iv) house care support for patients with special needs, (v) collaborative acts between ministries such as the Ministry of Health, the Ministry of Education, the Ministry of Labor and Social Security, and the Ministry of Family and Social Services (15). Additionally, in 2020 the Ministry of Health established the Autism, Mental Special Needs, and Rare Diseases Department (16), which aims to follow up on the diagnosis, treatment, and prognosis of patients with rare diseases and special needs. Although there is an apparent effort, the rare disease studies in Türkiye are still not at the desired level due to limited public awareness, limited numbers of special working groups, and a lack of national action plans on rare diseases.

Herein, we present patient and family-oriented survey data, which was held under a work package of the project named “ISTisNA-(IS*tanbul* T*anisiz ve* NA*dir Hastaliklara Çözüm Platformu*; Istanbul Solution Platform for Undiagnosed and Rare Diseases). In this regard, we held a patient-oriented survey to define the common problems of the patients and their families in social/work/school life, the burdens caused by a rare disease, and their expectations. This study includes the analysis and interpretation of the survey responses of patients and their relatives. To the best of our knowledge, this is Türkiye's first and most comprehensive survey on rare diseases.

## 2. Materials and methods

This survey was held under the project ISTisNA. ISTisNA was a feasibility project conducted by Acibadem University in partnership with Istanbul University and the Health Institutes of Türkiye (TUSEB) and funded by the Istanbul Development Agency (ISTKA). The project's main aim was to create a sustainable model that provides solutions for the diagnosis and treatment of undiagnosed and rare diseases. The project also aimed to raise awareness of rare diseases, generate a platform for researchers and industry, increase the network of research biobanks in Istanbul and Türkiye on rare diseases, connect the scientific community with patient organizations and provide education for both parties.

### 2.1. Target population

The survey's target population was patients with a rare or undiagnosed disease and/or their parents, spouses, siblings, or relatives taking care of these patients. The diseases with a frequency of < 1/2,000 are grouped as rare and conditions that remained unknown despite all the available tests and medical evaluation are grouped as undiagnosed. Also, families suffering from a rare disease that is not yet molecularly diagnosed were included. The survey was extensively distributed at two different time points *via* patient organizations (Supplementary Table 2) in Türkiye. In the beginning of the survey, an expletory abstract was presented with the overall purpose of the study as well as the aim, content and the study design (Supplementary material 2). The anonymity of the data was safeguarded as there was no personal identifying details obtained from respondents including the age (age groups were indicated between 0 and 12 months, 1 and 10, 10 and 18, 18 and 30, and over 30 years.), who completed the online survey. The Participants were required to consent through the survey web link before starting the survey.

### 2.2. Development of the survey

**Figure 1:**
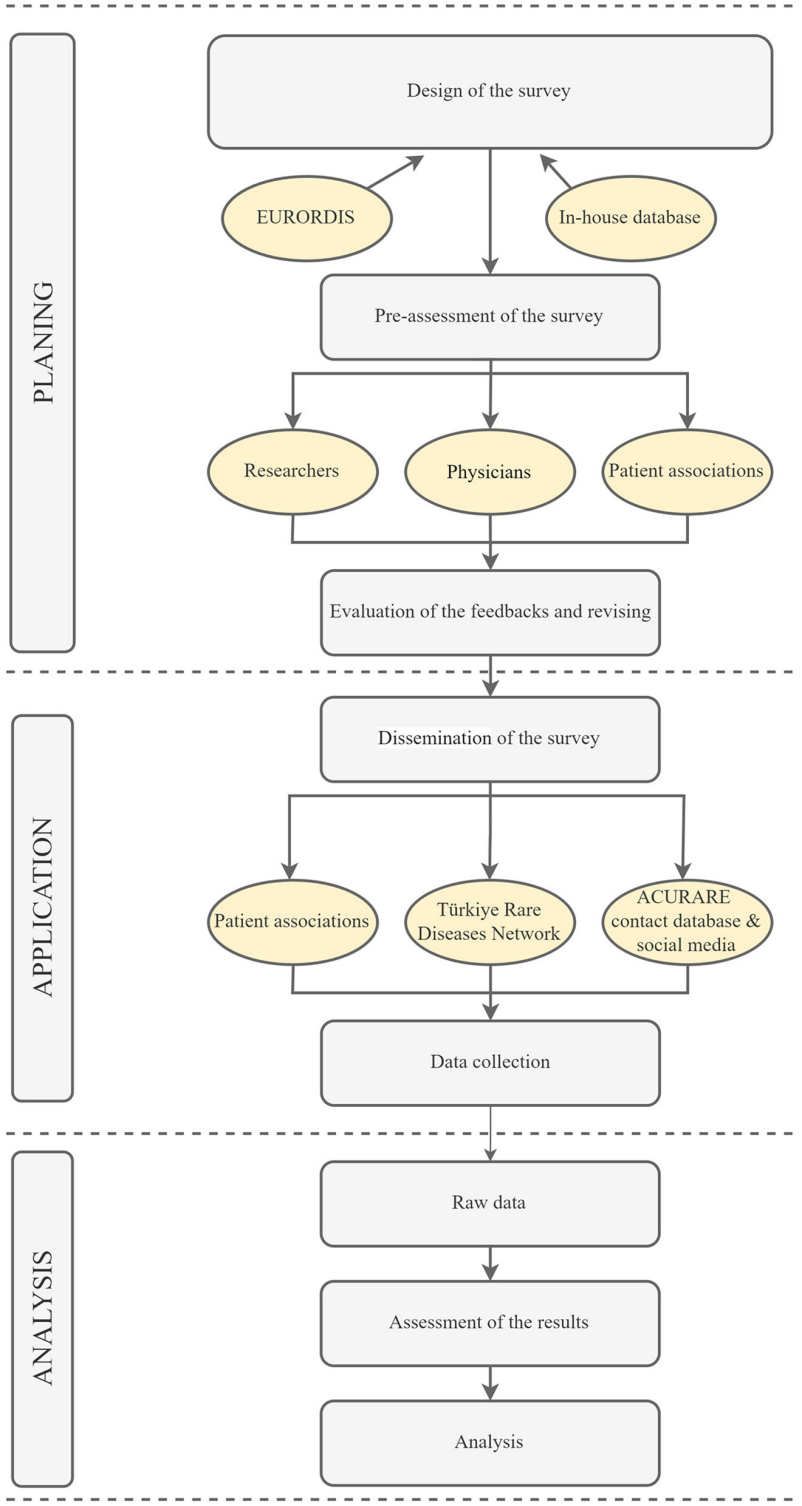
The experimental flow of the survey. The survey was constructed in three main steps: planning application and analysis. The questions were gathered from previously conducted surveys that are relevant for patients in Türkiye. Additional questions were created for country specific conditions. The dissemination of the survey was done through patient associations, Türkiye Rare Disease Network and ACURARE in-house contact databases, in addition to social media accounts of ACURARE, (Instagram: acuraretr, Twitter: acurare1, LinkedIn: ACURARE). The data were retrieved directly from the SurveyMonkey platform, exported to SPSS V21, and analyzed by the independent consultant service provider. The figure was created by Draw.io (https://drawio-app.com/).

The survey was developed based on literature, and different surveys from other regions of the world using the search terms “rare diseases + survey” on Pubmed (17–20). Eurordis' surveys were also translated into Turkish for relevant questions (21, 22). After selecting a wide range of questions, a team composed of medical doctors from different specialties (neurologists, pediatricians, pharmacologists, clinical geneticists, etc.), molecular geneticists, bioinformatics, patient organization representatives, and medical school and/or life sciences students were asked to evaluate the questions. A specialist in the language also assessed the survey to ensure that the questions were clear and understandable. A survey development software, Surveymonkey (23), was used to construct the survey. A total of 35 questions (one fill-in-the-blank) were selected. The survey was constructed in a way that, depending on the participants' answers, each participant would answer 30 questions in total, taking about 20–25 min. The workflow has been summarized in Figure 1.

The survey was constructed in different categories. The first part aimed to define the participants' profile (gender, age, and educational status) (patient or patient relative). Afterward, questions were asked to determine the current disease status (diagnosis, symptoms, etc.), experiences, challenges, and expectations.

Before distribution, the survey was subjected to a small group of individuals for a pilot test, including patient organization representatives, academicians, healthcare workers, students, rare disease researchers, and the non-scientific community. The survey was open between January 24 and February 22, 2020.

#### 2.2.1. Ethical governance

Ethical review and approval was not required for this study in accordance with the local legislation and institutional requirements. The participants provided their written informed consent to participate in this study.

### 2.3. Data analysis

The data were retrieved directly from the SurveyMonkey platform, exported to SPSS V21, and analyzed by the independent consultant service provider. The descriptive analysis included counts and percentages for each question. The 95% CIs were calculated for the single-choice questions. At the end of the 12 questions, the participants were given a space to respond in addition to the choices, and the results were presented in the manuscript where applicable. To avoid overlapping individuals the data was sorted by the diagnosis age of the patients and checked all the other answers. If there were more than 3 common answers (education, diagnosis, diagnosis age) the submitter would be omitted.

## 3. Results

In total, 498 individuals [caregivers (*n* = 416) and patients (*n* = 77), unknown (*n* = 5)] participated in survey, and the majority of the responders (76.3%, 95% CI: 72–80) were parents of individuals suffering from a rare or undiagnosed disease. This was followed by the patients themselves (15.6%, 95% CI: 13–19), a distant relative, friend, or physician.

### 3.1. Information on patients

The male-female ratio of the patients was almost 3:1, and 45% of the patients were in the age range of 1–10 years old (95% CI: 40–49), and 20% of them were in the age range of 10–18 (95% CI: 17–24). Of the patients aged 18–30 (*n* = 63), 35% were students; 27% were public, private employees, or self-employed; 28.6% were unemployed and/or not suitable for a job. Among patients older than 30 (*n* = 59), 54.2% were public, private, or self-employed. Consistent with the age group, the majority of patients in the survey were students (57.5%, 95% CI: 53–62), mainly in primary school or pre-school, followed by the unemployed and are not suitable for a job due to long-term illness and/or disability (17%, 95% CI: 14–21) (Table 1).

**Table 1:** The profile of the patients.

**Patient characteristics**	**% (95% CI)**
**Sex (*****n** =* **493)**	
Male	71.2 (67–75)
Female	28.8 (25–33)
**Age range (*****n** =* **495)**	
0–12 months	10.5 (8–14)
1–10 years	44.7 (40–49)
10–18 years	20.2 (17–24)
18–30 years	12.7 (10–16)
Over 30 years	11.9 (10–15)
**Educational background (*****n** =* **491)**	
Attending primary school	20.8 (17–25)
Preschool education age	19.8 (16–24)
Not graduated from any educational institution	15.5 (12–19)
High school graduate	12.2 (9–15)
Primary school graduate	11.8 (9–15)
Secondary school graduate	9.6 (7–13)
Bachelor's degree	6.1 (4–9)
Others (MSc^*^, Ph.D.^*^,AD^*^)	4.3 (3–6)
**Current occupation (*****n** =* **464)**	
Student	57.5 (53–62)
Unemployed and not eligible to work (long-term illness/disability)	17.0 (14–21)
An employee of the private sector	6.5 (4–9)
Housewife	6.5 (4–9)
An employee of public institutions	5.8 (4–8)
Others (Retired, Un^*^, SE^*^, long leave)	6.7 (5–10)
**Insurance Status (*****n** =* **49)**	
Social security institution (SSI)	81.8 (67–91)
Private health insurance	11.4 (4–25)
Supplementary insurance	4.5 (0.5–15)
No health insurance	2.3 (0.06–12)

^*^MSc, Master of Science; Ph.D., Philosophy of Doctorate; AD, associate degree; Un, unemployed; SE, self-employed; CI, Confidence Intervals.

In total, 91.1% (95% CI: 88–93) of the patients were clinically diagnosed and the most common disease among the participants was Duchenne/Becker muscular dystrophy (DMD/BMD, Table 2 and Supplementary Table 1) the average time to diagnosis was 6.0 months (min: 0–max: 546 months) for all age groups. There was no significant difference in the time to diagnosis between different age ranges among patients diagnosed with a disease. The 8.89% of the patients (*n* = 44, 25 male and 19 female) were still undiagnosed, and 15 of them were between the age range of 0–18, 13 were 18–30 and 16 were above 30 years old. Among undiagnosed patients older than 30 years, the mean age of onset was 22.8 years (min: 2, max: 54 years) and the age of onset of undiagnosed patients between the ages of 18–30 years started at the mean age of 7 years (min: 0, max: 20 years).

**Table 2:** The diagnoses of the survey participants.

**Diagnosis of the patients (*n =* 380)**	**% (95% CI)**
Duchenne Muscular Dystrophy/Becker Muscular Dystrophy (DMD)/(BMD)	55.8 (51–61)
Mucopolysaccharidosis (MPS, all types)	11.8 (9–16)
Cystic Fibrosis	8.7 (6–12)
Albinism (all types)	6.8 (5–10)
Bladder exstrophy	2.1 (1–4)
Cri-du-Chat syndrome	1.8 (1–4)
Systemic sclerosis-scleroderma	1.6 (1–3)
Familial Mediterranean Fever	1.1 (0.3–3)
Limb-Girdle Muscular Dystrophy (LGMD)	1.1 (0.3–3)
Retinitis Pigmentosa	1.1 (0.3–3)
Other^*^	8.2 (6–11)

In total 380 individuals answered the question and results above 1% are shown in table.

^*^All diagnoses are listed in Supplementary Table 1. CI, Confidence Intervals.

The patients' main caregivers were mothers (73%, 95% CI: 69–77). Fathers were mentioned as the primary caregiver in 15.5% of the participants (95% CI: 12–19), and only three participants stated that the care is shared between the mother and father. The average age of the primary caregivers fell into the 30–40 age range (46%, 95% CI: 42–51), and primary caregivers were mostly high school graduates (28%, %95 CI: 24–32). Only 35.3% (95% CI: 31–40) of caregivers declared an active working life, and 48% (95% CI: 43–52) of the caregivers were housewives (Table 3). Of the primary caregiver mothers (*n* = 353), 62.3% (95% CI: 57–67) were housewives and 24.4% (95% CI: 20–29) declared an active working life (public, private sector, freelance or non-governmental organizations).

**Table 3:** The profile of the patients' primary caregivers.

**Caregiver characteristics**	**% (95% CI)**
**(*****n** =* **492)**	
Mother	73.0 (69–77)
Father	15.5 (12–19)
Spouse	4.5 (3–7)
Others^*^	7.0 (5–10)
**Age range (*****n** =* **487)**	
18–30 years	16.6 (13–20)
30–40 years	46.0 (42–51)
40–50 years	27.1 (23–31)
50–60 years	6.8 (5–9)
Over 60 years	3.5 (2–6)
**Educational background (*****n** =* **483)**	
High school	28.2 (24–32)
Bachelor	19.5 (16–23)
Primary school	18.8 (15–23)
Secondary school	13.5 (11–17)
Associate degree	8.5 (6–11)
None	7.0 (5–10)
Master's degree	3.9 (2–6)
Ph.D. graduate	0.6 (0.1–2)
**Current occupation (*****n** =* **482)**	
Housewife	48.0 (43–52)
Public/private sector employees	28.6 (25–33)
Self–employed	6.0 (4–9)
Retired	5.2 (3–8)
Student	4.4 (3–7)
Unemployed and seeking a job	4.0 (2–6)
Others^**^	3.9 (2–6)
**Insurance status (*****n** =* **247)**	
Social security institution (SSI)	80.6 (75–85)
Private health insurance	3.24 (1–6)
Supplementary insurance	4.05 (2–7)
No health insurance	12.15 (8–16)

^*^Siblings or 2^nd^-degree relatives,

^**^not eligible to work, on a long leave, non-governmental organization. CI, Confidence Intervals.

The question regarding the insurance status was answered by 50% of the participants. According to the responses, more than 80% of the patients were members of the General Social Security Institution of Türkiye, while 31 (11%) stated that the patient did not have any health insurance.

### 3.2. Patients' experiences and needs during the disease process

To evaluate the patients' experiences, we designed the survey to assess the incidents under the topics: of psychology, work, education, disease process, and daily life.

#### 3.2.1. Psychological experiences

**Figure 2:**
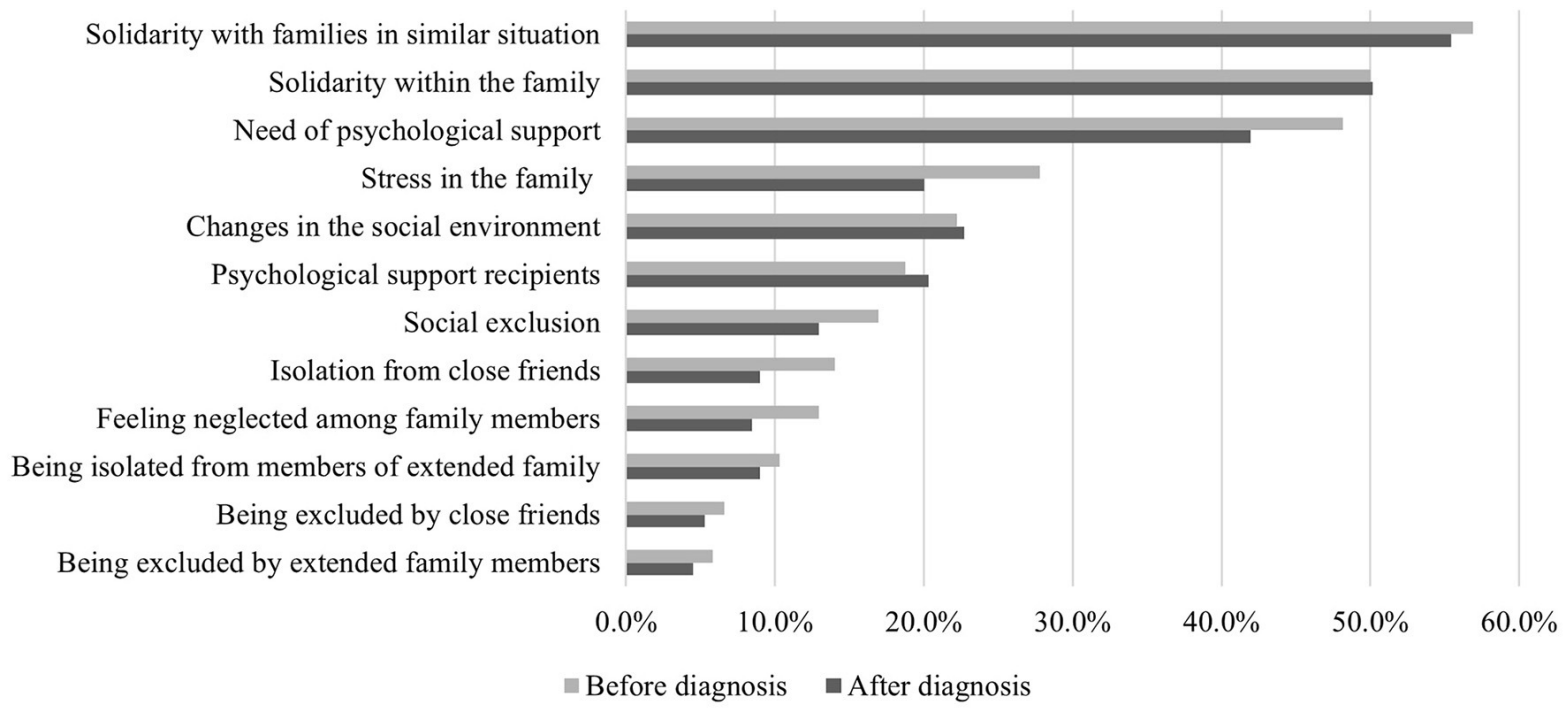
The changes in experiences before and after diagnosis. The experiences of the patients before and after the diagnosis were evaluated in two questions and the results were compared. A total of 378 participants answered the question and more than one option could be selected.

The need to reach families dealing with similar diseases and the need for solidarity was mentioned as the highest experience before (56.8%) and after (55.4%) having a diagnosis. This was followed by strengthening solidarity within the family with a rate of 50%. Approximately 48% of the participants stated that they needed psychological support, but only 18% of these received professional help before the diagnosis. Although not significant (*p* = *0.8*), we determined that the need for psychological support was lower among the patients/families after diagnosis and 20% of these went under psychological therapy. Additionally, the stress within the family caused by the ambiguity decreased after the diagnosis (Figure 2).

#### 3.2.2. Work and education

Almost 30% of the patients' caregivers had to quit working for the full-time needs of the patient (Table 4). Despite the challenges, most of the patients (75%) could continue to work or attend school. More than 60% of the patients declared that the school and/or workplace made special arrangements for patients' needs. These arrangements mainly consisted of changes in the classroom by the initiative of a teacher or school management. This was an open-end question, allowing participants to write about their experiences as well. They stated that; letting the mother accompany the patient at school, granting special permission for particular medical needs, providing flexibility in school attendance, and special attention from the teachers positively impact patients (Table 4). However, almost 40% of the patients declared that the school and/or workplace did not make and/or refused to make special arrangements for the patient's needs. Some issues like coming across with discrimination by the administrative workers and/or teachers, peer violence, and refusing to enroll disabled students at school had a negative impact on their academic life. The most common adverse experiences mentioned by the participants with an active job were discriminatory behaviors, mobbing, and refusal of disability reports by the managers.

**Table 4:** The impact of rare diseases on patients and caretakers.

**The impact of rare disease on …**	**% (95% CI)**
**… the work or education life (*****n** =* **408)**	
No	75.3 (71–79)
Yes	24.8 (21–29)
… work or education environment^*^(***n** =* **382)**	
No special changes	35.6 (31–41)
Changes within the classroom (Additional study hours, assistance in communicating with friends, etc.)	23.6 (19–28)
Special leave permit for the patient	14.4 (11–18)
Support for transportation (Service, vehicle, etc.)	12.6 (9–16)
Accessibility/equipment (elevators and special chairs)	11.8 (9–15)
A special change request was denied.	6.8 (4–10)
Other	30.4 (26–35)
**… caretakers work or education life (*****n** =* **404)**	
No	71.5 (67–76)
Yes	28.5 (24–33)
**…to be away from work or school (caretakers**, ***n** =* **236)**	
< 20 days	44.9 (38–52)
Quit from work / school	39.0 (33–46)
20–40 days	12.3 (8–17)
More than 70 days	2.5 (1–5)
41–70 days	1.3 (0.3–4)

The participants asked to fill in the blank sentence to explain the impact of a rare disease to their work/education life and/or environment.

^*^The participants were able to select minimum 3 and maximum 5 options.

#### 3.2.3. Disease process

Our survey also had a section to evaluate the experiences during diagnosis, treatment, and management processes. The open-end question evaluating the experiences before diagnosis was answered by almost all the participants. The main topics were lack of knowledge about the conditions, lack of medical specialists on the specific disease, long testing period or lack of centers for diagnosis, and need to travel to major cities such as Istanbul and Ankara to have a diagnosis, treatment, and/or management of the disease.

Another question evaluated the access rates to the diagnosis, medical specialists, hospitals, and testing. Almost 81% of the participants find it challenging to access a medical specialist, followed by access to treatments and medicines with 73% and access to hospital appointments and admissions with 50%. Almost 20% of the participants also mentioned difficulties reaching additional home care services (Table 5). Only 13.5% of the participants could receive public services, and 26% were attending private care centers from their budgets. Sixty percent of the patients stated that they received rehabilitation services and therapies (occupational therapy, speech, or physical therapy, etc.). The frequency of the patients receiving support for rehabilitation and treatment predominantly 1–2 hours per week was 39% (Supplementary Table 3).

**Table 5:** Difficulties in accession to public/private services.

**Which of the following options are the most difficult to access while dealing with the disease?**	**%**
Access to specialist physicians	80.7
Treatment and medications/supplements	72.8
Hospital admissions/appointments	50.0
Diagnosis	42.1
Equipment and investments (wheelchair, home modification, etc.)	41.7
Meetings with healthcare professionals	28.3
Tests and evaluations	26.2
Home care services (home assistance, personal assistant, caregivers)	19.7
Special nutrition	16.2
Municipal special services	10.3
Governorate/district governorship special services	8.6
Periodic maintenance services	8.3
Patient associations	5.5

The participants were able to select minimum 3 and maximum 5 options.

#### 3.2.4. Daily life

Another topic covered by the survey was the daily life problems encountered during the disease process (Table 6). The highest selected option was the daily life difficulties experienced at school (61%). These difficulties were followed by self-care activities such as hygiene, dressing, eating (56%), social life, and relationships with other people (52%), followed by inability to drive, exclusion from social life, and needing care.

**Table 6:** Major problems faced by the patient when dealing with a rare disease.

**What are the major problems faced by the patient when dealing with a rare disease?**	**%**
Difficulties experienced at school	60.7
Personal care activities (personal hygiene, dressing/undressing, eating, etc.)	55.2
Social life and relationships	52.4
Motor and sensory functions (vision, hearing, difficulties in body postures, etc.)	47.6
To reach school/work	36.6
Daily activities and tasks (chores, food preparation, shopping, etc.)	34.5
Being able to carry out daily work	23.8
Inability to manage financial situation/budget	18.6
Learning and understanding	16.2
General behavior control	15.9
Difficulties experienced in business life	12.8
Communication with others (participating in a chat, sending e-mail)	7.9

We also asked a fill-in-the-blank question: “To have a rare disease in our lives … “ and by far, the most preferred answer was the “*restriction of social life*” with 75.8%. Besides, approximately one-third of the participants mentioned a decreased income, restriction/cessation of professional activity, and disconnect from their entourage due to a rare disease (Supplementary Table 4).

### 3.3. Expectations

The survey also aimed to reveal the expectations of the individuals affected by a rare disease. Almost all participants stated the need for medical specialists to be aware of rare diseases and their management. Around 75% of the participants also mentioned the lack of information and resources, especially in Turkish. More than 60% of the participants were willing to be informed about the ongoing research activities on rare diseases, and 50% of these would be willing to participate in a clinical trial if they had detailed information (Table 7; Supplementary Table 5).

**Table 7:** Priorities for the patient and patient families in diagnosis and treatment process.

**Which of the following is a priority for the patient and patient families in the diagnosis and treatment processes?**	**%**
Reaching the right specialist physician	93.8
Accessing the accurate source of information about the disease	73.5
To be aware of new research studies on the disease	62.4
Access to medication	58.6
Reaching similar patients and/or patient support groups	49.4
Supply special support devices (battery-powered chairs, hearing aids, special glasses, other daily orthopedic devices) and specific treatments (orthodontic, etc.)	39.3
Reaching the right healthcare professionals	30.7
Home care services	17.6
In your opinion, which of the following options would be more useful to overcome obstacles during diagnostic and treatment process?	
The establishment of rare disease-specific diagnostic and treatment centers	89.0
Access to specialist medical care and treatment	57.9
Reducing bureaucracy in treatment processes	51.7
Expanding awareness of healthcare professionals about rare diseases	44.1
Comprehensive support for rare diseases, such as the provision of social care, special education support	43.5
Finding interfaces that will provide access to current and accurate information in the field of rare diseases	35.5
Providing consultancy to reach the accurate physicians and healthcare professionals	26.6
Improving the communication of healthcare professionals with people with rare diseases/ patient relatives	26.5
Overcoming difficulties in transitions between pediatric and adult clinics	24.8
Coordination between family physician and hospital care	19.0
Coordination between health services and social care services	18.3
Informing patients' relatives/health professionals about orphan drugs	15.2

The survey also directed a question to the participants for their opinion on overcoming the obstacles in rare diseases. Almost 90% mentioned the need to establish diagnosis and treatment centers specialized in rare diseases. This was followed by reducing bureaucracy in treatment processes by 52%. Survey respondents also stated the need for the necessary equipment for the disease and all disease-related drugs/supplements to be provided free of charge by the General Social Security Institution of Türkiye (Table 7). Finally, yet importantly, the participants also mentioned the need to obtain detailed information from healthcare professionals and physicians.

## 4. Discussion

Studies on rare diseases are still developing in Türkiye. Despite the recently increased activities of the state, the late-starting interest has decreased with additional restrictions due to the COVID-19 pandemic, as is the case elsewhere in the world. There is a limited number of studies, evaluating the impact of rare diseases on families and none was conducted in Türkiye (21, 24). Here, we aimed to pinpoint the current situation of the patients, the obstacles they come across with, and their expectations through a comprehensive survey.

One of the most critical problems for individuals with rare diseases and their families is the difficulty of early and definitive diagnosis. An unprecedented proportion of the patients in our study were diagnosed at the time of the first symptoms, but this can be explained by the fact that most participants were affected by well-described diseases, such as albinism, DMD/BMD, cystic fibrosis (CF), which are relatively easy to diagnose by first symptoms and/or newborn screening tests. In our respondents, almost 10% of the patients remained undiagnosed, around the same range as previous findings (6)(25). Recently, we have received the approval of the continuation project of ISTisNA by Istanbul Development Agency. The new project has been designed according to the results of this survey. One of the work packages is establishment of Undiagnosed Disease Program. In this program we will be setting up an online platform in which the physicians can apply with the clinical, laboratory and molecular test of their undiagnosed patients. The program will setup a multidisciplinary council and evaluate the patients and suggest additional tests or analysis to solve the diagnosis.

Prolonged diagnosis time with a rare disease also strains the patient psychologically. Often patients and their families experience a sense of isolation, injustice, and uncertainty about their future (26). It was previously shown that individuals with rare diseases experience more depression, anxiety, and stress than the general population (27), and even if their symptoms are similar, people with rare diseases are at greater risk of depression and anxiety than people with common diseases (28). In a recent study from Türkiye, the researchers also declared that there was a significant decrease in the anxiety levels of the families after diagnosis, but this was not reflected in trait anxiety levels (29). The Australian study declared that rare diseases had a moderate impact on the families with a wide range (24). Our results on the psychological needs before, during, and after the diagnosis revealed that the need for professional support decreased after the patient received a diagnosis. This confirms that being diagnosed significantly reduces anxiety among patients and their family members.

The parents of a rare disease patient experience an enormous change in their lives in parallel to the scope of the responsibilities they take. In Türkiye, the primary caregivers of the patients are primarily mothers (30). In addition, an unprecedented number of caregivers stated that they were deprived of their work or academic life to provide full-time care to the patient. Most caregivers cannot receive any support for housework and/or daily errands in the patient's household. These results show that most mothers carry the burden of the rare disease and household/daily chores and make sacrifices that will change their lives enormously. This puts women away from an active social and working life and lowers the house's income.

Rare diseases show distinct clinical, diagnostic, and/or management requirements, and rearrangements are needed in school and work environments to improve their quality of life. In Türkiye, the rights of disabled individuals are protected by the law, but these regulations do not always cover all the necessities of rare diseases. According to the findings of this study, these arrangements were made by individual initiatives of the responsible personnel, which were often inadequate and/or incompatible.

The number of rare disease centers in Türkiye is still not at the required level, and the experienced physicians reside in more central cities like Istanbul, Ankara, and Izmir. This issue forces patients to travel to these cities for proper treatment and healthcare. According to the “EURORDISCARE 2” survey results, 25% of patients had to travel to a different region to receive a confirmatory diagnosis (21). This was repetitively mentioned in the answers to the open-end questions in our survey, which leads us to the assumption that the rate is also high in Türkiye. Although the patients must go through a challenging process to receive appropriate treatment, in Türkiye, general health insurance covers an essential part of the diagnosis and disease management. Every citizen, including the immigrants, under 18 years old, is supported by standard state insurance. As in Australia, Turkish government provides financial support to families with disabled child but both studies showed that this support was not enough to cover all the medical expenses (24). Easing the administrative processes for disability reports and/or prescriptions should be a priority for Türkiye's effective rare disease act. Patients also stated that they had difficulty accessing treatment and drugs while coping with the disease. This finding is consistent with a recent study examining patients' access to orphan drugs in Türkiye. In this context, access to 34 of the 105 orphan drugs on the EMA list in 2020 is still not available, and only 34 of the remaining 71 drugs are within the scope of reimbursement (31).

Research on rare diseases are already in the national plans of European countries starting from France, Bulgaria, Greece, Portugal, and Spain (32). This action plans also lead to set up major goals like (i) all patients should be diagnosed within 1 year and (ii) all undiagnosed individuals should be enrolled to a globally coordinated diagnostic and research pipelines (33). In our study, the majority of the responders were willing to attend to research activities if they were well informed. This finding is promising since, it is essential to promote not only medical treatment but also research activities for undiagnosed and rare diseases. The research activities should be handled in a holistic manner including biobanking, advanced NGS applications, functional studies and clinical trials. Two of the many aims of the continuation project of ISTisNA are integration of Biobanks in Istanbul and trio whole genome sequencing of undiagnosed patients who were enrolled to ISTisNA-Undiagnosed Disease Program.

Rare diseases are often difficult to notice; they present heterogeneous symptoms that features of common diseases can mask. The highest expectations of the patients in Türkiye are the establishment of specialized centers on rare diseases and more educational activities targeting healthcare providers, which would be beneficial in eliminating the problems encountered with the diagnosis and treatment process. There is also a lack of information published in Turkish, and people expect to reach essential booklets and explanatory documents for their conditions. In this regard, Acibadem University Rare Diseases and Orphan Drugs Application and Research Center (34) initiated the translation of disease information provided on the Orphanet website into Turkish. We first translated the first 149 diseases, which cover almost 75 to 80% of the population burden of rare diseases (35). Up to date, 1007 diseases have already been translated into Turkish and uploaded to the ACURARE website (36). Moreover, within the scope of ORPHANET-TR activities, with the leadership of TUSEB, National Orphanet Advisory Board performed an Orphanet nomenclature translation of 11,000 diseases registered on the Orphanet website. The most critical impact of this initiation will be to support the integration of rare diseases into the General Social Security System for diagnosis, treatment, management, and, most importantly, reimbursement of drug costs. Secondly, it will allow a harmonization of the disease-specific ORPHA codes to be implemented in the system, which will provide an international level of standardization within the Turkish Social Security System (37).

There were also several limitations of the study. Firstly, the distribution of the survey was done *via* patient associations, which limited the represented disease groups. Although the number of participants was quite high, each participant did not answer all questions, which limited the analysis. Another limitation of the study was due to the participants' diagnosis. More than 75% of the responses were coming from patients/families with DMD/BMD, MPS and CF associations, which made it difficult to perform a disease subgroup analysis.

This survey supports the findings that have been published previously from other countries as well (21, 24, 38, 39). Rare disease communities, regardless of nationality, share the same type of difficulties and needs:

- lack of information about rare diseases, which leads to prolonged or misdiagnosis, and inability to access detailed information about the disease,
- lack of specialist physicians and diagnostic centers,
- long duration of tests and treatments,
- financial and psychological difficulties created by all these processes.

To our knowledge, this is the most comprehensive set of questions answered by an exceptional number of rare disease patients and/or their families in Türkiye. We need more of these studies regarding rare diseases to determine the priorities of the field in our country and these activities should be taken by the state in a much broader approach. This survey is one of the many activities run by the feasibility project ISTisNA. In the newly approved ISTisNA project, our aim is to create a sustainable model to create solutions that are pointed out in this study. We believe these findings will provide a starting point to determine the primary obstacles of rare diseases in Türkiye and a guide to take immediate action.

## Data availability statement

The raw data supporting the conclusions of this article will be made available by the authors, without undue reservation.

## Ethics statement

Ethical review and approval was not required for this study in accordance with the local legislation and institutional requirements. The participants provided their written informed consent to participate in this study.

## Author contributions

OH: survey creation and conducting, writing original draft, data analysis, and supervision. ISah: survey creation, writing original draft, data conducting, and analysis. YE: survey creation, conducting, and review and editing. OO: data analysis, review, and editing. EY: survey creation, review, and editing. MY and NE: dissemination and editing. OA: survey creation, review, and editing. SU: survey creation, conducting, review, and editing. YA: survey creation, review, and editing. ISat: survey creation, review, and editing. UO: supervision, funding acquisition, survey creation, review, and editing. All authors read and approved the final manuscript.
