# Supplementary material for: Obstacles and expectations of rare disease patients and their families in Türkiye: ISTisNA project survey results
Source: Front Public Health. 2023 Jan 4;10:1049349. doi: 10.3389/fpubh.2022.1049349 (PMC9846031; doi:10.3389/fpubh.2022.1049349)
Supplement: Supplementary file 1 [file Data_Sheet_1.PDF]

Supplementary Material

Supplementary Table 1. List of all patients' diagnoses

**Diagnosis of the patients (n=380)**	**% (95% CI)**
DMD/BMD	55.8 (51.0-61.0)
Mucopolysaccharidosis (MPS, all types)	11.8 (9.0-16.0)
Cystic Fibrosis	8.70 (6.0-12.0)
Albinism	6.80 (5.0-10.0)
Bladder exstrophy	2.10 (1.0-4.0)
Cri-du-Chat syndrome	1.80 (1.0-4.0)
Systemic sclerosis-scleroderma	1.60 (1.0-3.0)
Familial Mediterranean Fever	1.10 (0.3-3.0)
Limb-Girdle Muscular Dystrophy	1.10 (0.3-3.0)
Retinitis Pigmentosa	1.10 (0.3-3.0)
Down syndrome	0.80 (0.2-2.0)
Pulmonary hypertension	0.80 (0.2-2.0)
Fabry disease	0.50 (0.06-2.0)
Rett syndrome	0.50 (0.06-2.0)
Cystinosis	0.50 (0.06-2.0)
Williams syndrome	0.50 (0.06-2.0)
1q24 deletion syndrome	0.30 (0.01-1.0)
Ataxia-telangiectasia	0.30 (0.01-1.0)
Crohn's disease	0.30 (0.01-1.0)
Desbuquois syndrome	0.30 (0.01-1.0)
Eisenmenger syndrome	0.30 (0.01-1.0)
Kabuki Syndrome	0.30 (0.01-1.0)

Congenital muscular dystrophy	0.30 (0.01-1.0)
Lennox–Gastaut syndrome	0.30 (0.01-1.0)
Ohtahara syndrome	0.30 (0.01-1.0)
Osteogenesis imperfecta	0.30 (0.01-1.0)
Osteopetrosis	0.30 (0.01-1.0)
Primary Ciliary Dyskinesia	0.30 (0.01-1.0)
Spinal muscular atrophy	0.30 (0.01-1.0)
Tay-Sachs disease	0.30 (0.01-1.0)
Treacher Collins syndrome	0.30 (0.01-1.0)
Thrombotic thrombocytopenic purpura	0.30 (0.01-1.0)
ZTTK Syndrome	0.30 (0.01-1.0)

DMD/BMD: Duchenne Muscular Dystrophy/Becker Muscular Dystrophy. CI: Confidence Intervals.

Supplementary Table 2. Associations under Rare Disease Network and their number of members.

Association Name (English)	Registered Members	Social Media Members (FB, Instagram, LinkedIn etc.)	URL
MPS-LH Association- Mucopolysaccharidosis Lysosomal Storage Diseases Association	850	9335	https://mpsturk.org/
Cystic Fibrosis Family Assistance and Solidarity Association	380	20934	http://www.kifder.org.tr/
Pulmonary Hypertension and Scleroderma Patient Association	300	6409	https://www.pahssc.org.tr/
Albinism Association	198	4691	https://www.albinizm.org.tr/
Achondroplasia and Family Association	83	1081	https://akondroplazi.org.tr/
Türkiye Spinal Muscular Atrophy Association	70	31015	https://www.sma.org.tr/
Duchenne muscular dystrophy Family Association	63	17960	https://www.dmdaileleri.org/
Rare Autoimmune Rheumatic Diseases Solidarity Association	55	7749	http://www.faromder.org/
Williams Syndrome Association	48	1263	No available web site
Phenylketonuria Family Association	45	12365	https://www.pkuaile.com/
Association for Life with Ataxia Telangiectasia	42	1551	https://atileyasam.org/
Retinitis Pigmentosa Patients Platform	110	4200	https://www.facebook.com/groups/166960586675729/?ref=share
Cystinosis Patients Association	79	1273	https://www.sistinder.org/
Tay-Sachs and Sandoff Charity Association	25	1133	https://www.instagram.com/taysachsandhoff/
Happy With My Face Association	43	13300	www.yuzumlemutluyum.org.tr/
Total	2391	**134259**	

Supplementary Table 3. Additional support for patient care

Supportive individuals/services for additional patient care (n=289)*	**%**
Parent	64.0
Family member	33.6
Private care service from personal budget	26.6
None of them	21.5
Social or community services	13.5
Friends	6.2
Private care service from patient organizations	3.1
Average hours of support per week for rehabilitation services and therapies (n=284) **	**%**
No support	39.9
1-2 hours	38.5
3 - 4 hours	15.3
5 - 8 hours	4.2
9 - 12 hours	1.0
More than 12 hours	1.0

*****Participants could select more than one option, ******occupational therapy, speech, or physical therapy, etc.

Supplementary Table 4. Impact of the rare disease on patient's lives

The rare disease into our lives … (n=244)*	**%**
Restricted our social life.	75.8
Caused our revenue to decline.	41.8
Caused us to reduce / stop our professional activity.	36.1
Caused us to disconnect from our entourage	34.8
Limited our professional choices.	32.4
Limited our employment opportunities.	27.1
Prevented our access to higher levels of education.	17.6
Limited promotion opportunity.	11.1

*****Participants could select more than one option.

Supplementary Table 5. Patients' perceptions of participating in clinical trials.

Would the patient consider participating in research studies on information gathering about the disease?* (n=411)	**% (95% CI)**
After receiving detailed information about the study, it may be.	44.5 (40-49)
Yes.	43.6 (39-49)
No.	11.9 (9-15)
Would the patient consider participating in clinical trials on the disease?* (n= 410)	**% (95% CI)**
After receiving detailed information about the study, it may be.	50.8 (46-56)
Yes.	39.0 (34-44)
No.	10.2 (7-14)

* Does the patient’s relative give consent for the patient to participate in research for information gathering (If a guardian was appointed)?
