# Supplementary material for: Obstacles and expectations of rare disease patients and their families in Türkiye: ISTisNA project survey results
Source: Front Public Health. 2023 Jan 4;10:1049349. doi: 10.3389/fpubh.2022.1049349 (PMC9846031; doi:10.3389/fpubh.2022.1049349)
Supplement: Supplementary file 2 [file Data_Sheet_2.PDF]

ISTisNA Platform - Patient / Patient Relative Questionnaire

ISTisNA – Istanbul Undiagnosed and Rare Diseases **Solution Platform *Questionnaire***

This survey has been prepared within the scope of the "**ISTisNA – *Istanbul Undiagnosed and Rare Diseases Solution Platform Feasibility Project",*** **which is jointly carried out by Acibadem University, Istanbul University and TÜSEB and supported by Istanbul Development Agency 2019 Feasibility Support Program. ISTisNA – Istanbul Undiagnosed and Rare Diseases Solution Platform Feasibility Project will determine the roadmap of the ISTisNA Platform, which aims to produce solutions for the diagnosis and treatment of an important disadvantaged group such as undiagnosed and rare diseases.**

Participation in the survey we conducted in this context is on a voluntary basis. Although the number of questions in the questionnaire varies depending on your answers, it has been determined as approximately 30. The survey takes approximately 20 minutes to complete.

The questions that must be answered in the questionnaire are marked with (*). If these questions are skipped or the required number of options is not ticked, you will be automatically returned to the relevant question at the end of the survey. After answering the question / ticking the required number of options, you can complete the survey by going directly to the bottom of the page.

Your answers to the questionnaire will only be used in the Feasibility Report of İSTisNA – Istanbul Undiagnosed and Rare Diseases Solution Platform.

Thank you very much for your participation and support in our survey.

1. Who is filling out the questionnaire?

- The patient herself/himself
- Patient relative (Spouse)
- Patient relative (Parent)
- Patient relative (Grandparent)
- Patient relative (Sibling)
- Patient relative (Aunt, Uncle, etc.)
- Other (please specify)

2. Who is the most responsible person for the patient's care? (This person will be referred to as the **patient relative** in the continuation of the questionnaire.)

- Mother
- Grandmother
- Father
- Grandfather
- Sister
- Aunt
- Brother
- Uncle
- Spouse
- Other (please specify)

3. Is the person specified as the patient relative the person who is filling out the questionnaire?

- Yes
- No

4. What is the gender of the patient?

- Female
- Male

5. What is the patient's age?

- 0 - 12 months
- 18 - 30 years old
- 1 - 10 years old
- over 30 years old
- 10 - 18 years old

6. What is the age of the patient relative?

- 18 - 30 years old
- 50 - 60 years old
- 30 - 40 years old
- over 60 years old
- 40 - 50 years old

7. What is the patient's educational status? (If still a student, please tick the last graduated educational institution.)

- Not graduated from any educational institution.
- In pre-school age
- Continues to primary school
- Elementary / primary school graduate
- Middle school graduate
- High school graduate
- Pre-bachelor’s degree graduate
- Bachelor’s degree graduate
- Master’s degree graduate
- PhD graduate

8. What is the education level of the patient relative? (If still a student, please tick the last graduated educational institution.)

- Not graduated from any educational institution
- Elementary school / primary school
- Middle School
- High school
- Pre-bachelor’s
- Bachelor’s
- Master’s
- PhD

9. What is the current job status of the patient?

- Student
- private sector employee
- public institution employee
- freelancer
- Housewife
- Retired
- On long leave
- Unemployed and unfit to work (long-term illness/disability)
- Unemployed and looking for a job

10. What is the current job status of the patient relative?

- Student
- private sector employee
- public institution employee
- freelancer
- non-governmental organization employee
- Housewife
- Retired
- On long leave
- Unemployed and unfit to work (long-term illness/disability)
- Unemployed and looking for a job

11. How old was the patient when consulted to doctor for symptoms of the disease? (Write as a number)

12. Could the diagnosis of the disease be made?

- Yes
- No

ISTisNA Platform - Patient / Patient Relative Questionnaire

13. How old was the patient when the diagnosis was made?

14. What is the diagnosis of the disease?

15. What were the difficulties you experienced during the diagnosis process?

16. Does the patient consider participating in research to collect information about his disease?

(in case of guardian)

Does the patient's relative give permission for the patient to participate in research for information gathering purposes?

- Yes
- No
- Maybe after getting detailed information about the research.

17. Does the patient consider participating in clinical research on his disease?

(in case of guardian)

Does the patient's relative give permission for the patient to participate in clinical trials?

- ○ Yes
- ○ No
- ○ It can be after getting detailed information about the research.

18. ***Has the family experienced any of the following situations during the period from the onset of the disease symptoms to the diagnosis of the disease?*** (More than one option can be ticked.)

- ☐ Feeling of neglect among family members
- ☐ Tension between family members
- ☐ Being isolated from extended family members
- ☐ Exclusion by extended family members
- ☐ Strengthening solidarity within the family unit
- ☐ Divorce or separation
- ☐ Being isolated from close friends
- ☐ Being ostracized by close friends
- ☐ Social exclusion
- ☐ Reaching and solidarity with families in similar situations
- ☐ Change of social environment
- ☐ Needing psychological help
- ☐ Getting psychological help

Other (please specify)

19. ***In the process that started after the diagnosis of the disease,*** did the patient experience any of the following situations? (More than one option can be ticked.)

- ☐ The disease has not been diagnosed yet.
- ☐ Feeling of neglect among family members
- ☐ Tension between family members
- ☐ Being isolated from extended family members
- ☐ Exclusion by extended family members
- ☐ Strengthening solidarity within the family unit
- ☐ Divorce or separation
- ☐ Being isolated from close friends
- ☐ Being ostracized by close friends
- ☐ Social exclusion
- ☐ Solidarity with families in similar situations
- ☐ Change of social environment
- ☐ Needing psychological help
- ☐ Getting psychological help

Other (please specify)

20. Has the patient been deprived of work or education due to illness?

- ◯ Yes
- ◯ No

21. Have specific changes been made to the patient's work or school environment through one or more of the following options? (More than one option can be ticked.)

- ☐ Job duties / working hours have been rearranged. (more flexible hours, change of responsibility etc.)
- ☐ Changes were made in the classroom at the initiative of the teacher/ school. (additional study hours, help in communicating with friends, etc.)
- ☐ Special permission has been granted for rare disease.
- ☐ Dedicated support provided during arrival/departure. (service, vehicle, etc.)
- ☐ Other (please specify)
- ☐ Equipment and accessibility such as elevators and special chairs were provided.
- ☐ No special changes were made.
- ☐ The request for a special change was denied.

22. Did the patient relative have to quit his job or school due to illness?

- ◯ Yes
- ◯ No

İSTANBUL KALKINMA AJANSI

T.C. SANAYİ VE TEKNOLOJİ BAKANLIĞI

İSTisNA
İstanbul Tanısız ve Nadir Hastalıklara Çözüm Platformu

ISTisNA Platform - Patient / Patient Relative Questionnaire

23. What is the reason for the patient relative to change or leave the job after the symptoms of the disease appear?

- Decided to take care of the patient full time.
- Decided to work part time/free time job.
- Dismissed or sent.
- Left due to compulsory duty. (military service, compulsory service, etc.)
- Other (please specify)
- Contract has expired.
- Had to retire early.
- Retired.

24. What is the frequency of the patient's relatives being unable to go to work or school due to the requirements related to the patient's care?

- less than 20 days
- 20 – 40 days
- 41 – 70 days
- more than 70 days
- He left work/school completely.

25. Indicate the working type of the patient relative.

- ◯ Full-time fixed-contract
- ◯ Part-time fixed-contract
- ◯ Full-time contracted
- ◯ Part-time contracted
- ◯ Flexible
- ◯ Unemployed
- ◯ Other (please specify)

26. What is your insurance status?

- ☐ SSI
- ☐ Private insurance
- ☐ No health insurance
- ☐ Supplementary insurance

27. Complete the following sentence in the most appropriate way for you. (More than one option can be ticked.)

The introduction of a rare disease into our lives...

- ☐ limited our professional choices.
- ☐ Causes us to reduce/stop our professional activity.
- ☐ caused a decrease in our income.
- ☐ limited our employment opportunities.
- ☐ limited our opportunity for promotion.
- ☐ has prevented our access to higher education.
- ☐ has limited our social life.
- ☐ caused us to break away from our circle of friends.

Other (please specify)

* 28. Which of the following options is most difficult to reach when coping with the disease? **(Minimum 3, maximum 5 options should be ticked.)**

- ☐ Diagnosis
- ☐ Treatment and medications
- ☐ Hospital check-ins/appointments
- ☐ Tests and evaluations
- ☐ Special nutrition
- ☐ Interviews with healthcare professionals
- ☐ Access to specialist physicians who recognize the disease
- ☐ Home care services (home assistance, personal assistant, caregivers)
- ☐ Devices and investments (wheelchair, home adaptation, etc.)
- ☐ Term-based care services
- ☐ Municipal special services
- ☐ Governor's / District Governor's special services
- ☐ Patient associations

29. In the current situation, which of the following persons or services are providing additional assistance for the care of the patient? (More than one option can be ticked.)

- [ ] Mother-father
- [ ] Family member
- [ ] Friends
- [ ] Private care service from your own budget
- [ ] Care service provided by social or community services (public)
- [ ] Care service provided by patient associations
- [ ] None

30. In the household where the patient lives, how many hours per week on average is support received for housework and daily work?

- ○ No support is received.
- ○ 12 hours
- ○ 3 - 4 hours
- ○ 5 - 8 hours
- ○ 9 - 12 hours
- ○ more than 12 hours

31. On average, how many hours of support are received per week for rehabilitation services and therapies (occupational therapy, speech or physical therapy, etc.)?

- ○ No support available.
- ○ 12 hours
- ○ 3 - 4 hours
- ○ 5 - 8 hours
- ○ 9 - 12 hours
- ○ more than 12 hours

* 32. What are the most important problems faced by the patient when coping with a rare disease? ***(Minimum 3, maximum 5 options should be ticked.)***

- [ ] Daily activities and tasks (housework, meal preparation, shopping, etc.)
- [ ] Motor and sensory functions (vision, hearing, difficulties in body postures, etc.)
- [ ] Personal care activities (personal hygiene, dressing/undressing, eating, etc.)
- [ ] Social life and relationship with others
- [ ] Difficulties in school
- [ ] Difficulties in business
- [ ] Other (please specify)
- [ ] General behavioral control
- [ ] Ability to manage financial situation / budget
- [ ] Ability to carry out daily tasks
- [ ] Understanding and learning
- [ ] Connecting with other people (joining a chat, sending an email)
- [ ] Going to school/work and returning etc.

* 33. Which of the following is a priority for the patient and his family in the diagnosis and treatment processes? ***(Minimum 3, maximum 5 options should be ticked.)***

- [ ] Reaching the right physicians
- [ ] Reaching the right allied health personnel
- [ ] Access to the right source of information about the disease
- [ ] Reaching similar patients / relatives
- [ ] Reaching the right patient support groups
- [ ] Supply of wheelchairs, hearing aids, special glasses, other daily orthopedic support devices and special treatments (orthodontic etc.)
- [ ] home care services
- [ ] Access to medicines
- [ ] Be aware of new research studies on the disease
- [ ] Other (please specify)

* 34. Which of the following options would be more beneficial in your opinion in order to eliminate the problems experienced during the diagnosis and treatment process? ***(Minimum 3, maximum 5 options should be ticked.)***

- [ ] Access to specialist, medical care and treatment
- [ ] Reducing bureaucracy in treatment processes
- [ ] Ensuring coordination between family, doctor and hospital care
- [ ] Coordination between health services and social care services
- [ ] Establishment of special diagnosis and treatment centers for rare diseases
- [ ] Eliminating the difficulties experienced in the transition between pediatric and adult clinics
- [ ] Finding interfaces that will provide access to up-to-date and accurate information in the field of rare diseases
- [ ] Raising awareness of healthcare professionals for rare diseases
- [ ] Providing consultancy to reach the right physicians and healthcare professionals
- [ ] Informing the relatives of patients about orphan drugs
- [ ] Developing the knowledge of health professionals about orphan drugs
- [ ] Improving the communication of healthcare professionals with rare disease owners
- [ ] Improving the communication of healthcare professionals with patients' relatives
- [ ] Availability of comprehensive support such as social care provision, special education support for rare diseases
- [ ] Other (please specify)

* 35. ISTisNA - Which services should be provided by the Istanbul Undiagnosed and Rare Diseases Solution Platform in the field of rare diseases? ***(Minimum 3, maximum 5 options should be ticked.)***

- [ ] Providing information about patient support groups and directing patients to support groups
- [ ] Providing awareness and training services for patient associations
- [ ] Providing information about treatment and care opportunities
- [ ] Providing training services to healthcare professionals (physicians, nurses, therapists, etc.)
- [ ] Providing training services to support personnel (maintenance personnel, RAM workers, local services workers, etc.)
- [ ] Providing education services to patients' relatives
- [ ] Other (please specify)
- [ ] Providing information about local services that can help patients and their relatives
- [ ] Provide information about your rights regarding rare diseases
- [ ] Providing information about the social and economic support you can get
- [ ] Announcement of new research
- [ ] Conducting research activities for undiagnosed and rare diseases
